# MEFV mutation frequencies in a Turkish cohort with low prevalence of familial Mediterranean fever

**DOI:** 10.3906/sag-2009-119

**Published:** 2021-05-30

**Authors:** Necati ÇAKIR, Hülya AZAKLI, Duran ÜSTEK, Ömer UYSAL, Eren GÖZKE

**Affiliations:** 1 Department of Internal Medicine, Faculty of Medicine, Istanbul Medeniyet University, İstanbul Turkey; 2 Department of Genetics, Institute for Experimental Medicine, İstanbul University, İstanbul Turkey; 3 Department of Statistics, Bezmialem Vakıf University, İstanbul Turkey; 4 Department of Neurology, Fatih Sultan Mehmet Teaching and Research Hospital, University of Health Sciences, İstanbul Turkey

**Keywords:** Familial Mediterranean fever, mutations, MEFV gene

## Abstract

**Background/aim:**

Familial Mediterranean fever (FMF) is a genetically recessive autoinflammatory disease caused by mutations in the Mediterranean fever (MEFV) gene. The aim of this study was to investigate the frequencies of the most common MEFV mutations among a sample of healthy individuals from the Havsa population of European Turkey, where FMF is less prevalent compared to Asian Turkey.

**Materials and methods:**

The study group consisted of 263 unrelated healthy adults. All of the participants were analyzed for the M694V, V726A, M680I, and E148Q mutations in the MEFV gene.

**Results:**

In total, 25 of the 263 individuals carried MEFV mutations (9.5%). The observed allele frequencies were 1.5% for M694V (95% confidence interval [CI] 0.5-2.5), 2.6% for E148Q (95% CI 1.6-3.9), 0.5% for M680I (95% CI 0.0-1.1), and 0.0% for V726A. The frequencies of the M694V, M680I, and E148Q mutations were not significantly different from allele frequencies (approximately 20%) determined for other regions of Turkey where FMF is more prevalent.

**Conclusion:**

These data suggest that the positivity of the MEFV gene mutation tests have lower predictive value in a population with low FMF prevalence.

## 1. Introduction

Familial Mediterranean fever (FMF) is a hereditary autoinflammatory disease characterized by recurrent episodes of fever, peritonitis, pleuritis, arthritis, and/or erysipelas-like erythema. This condition is particularly common in populations with Mediterranean ancestry, including Arabs, Turks, Armenians, and Jews. Recently, patients with FMF from varying ethnic backgrounds, including Americans, Italians, and Japanese, with no obvious connection to any high-risk population have been reported. FMF is generally thought to be transmitted as an autosomal recessive trait [1,2].

The Mediterranean fever (MEFV) gene is associated with the pathophysiology of FMF and has been mapped to chromosome 16 p13.3. It is comprised of 10 exons and encodes a 781 amino acid protein called pyrin or marenostrin, which is expressed in granulocytes, monocytes, dendritic cells, and synovial, peritoneal, and skin-derived fibroblasts [3,4]. Pyrin regulates inflammatory response via activation of interleukin-1β (IL-1 β) and NF-kappa в. MEFV mutations cause dysfunction of the inflammasome complex, leading to excessive activation of IL-1β [5].

To date, more than 70 MEFV gene mutations have been reported. Five mutational alleles of MEFV, namely, M694V, V726A, M694I, and M680I in exon 10 and E148Q in exon 2, account for 70%-80% of FMF cases in Mediterranean countries [5]. Mutations in exon 10 are more prevalent among FMF patients compared to non-FMF individuals, whereas the variant E148Q is slightly more frequent in the general population compared to FMF patients [2].

According to previous studies, the 4 most common MEFV mutations among Turkish populations are M694V, V726A, M680I, and E148Q [6,7]. In the present study, we quantified the frequencies of these 4 MEFV alleles in healthy individuals from the Havsa region, where the prevalence of FMF was previously reported to be approximately 0.006% (95% CI 0.005-0.007) [8]. We compared our results with the previously reported frequencies of these mutations in different regions of Turkey where FMF is more prevalent.

## 2. Subjects and methods

We conducted this study in cohorts from the rural Havsa region and a suburban Havsa town where we had previously performed an epidemiological study [8]. Havsa is a Turkish town on the border of Greece and Bulgaria. A total of 263 healthy participants were recruited for this study (139 women, mean age 44.7 ± 13.7 years; 124 men, mean age 43.5 ± 14.8 years; and a female/male ratio 1.12). None of the participants had symptoms or a family history of FMF. The subjects were consecutively selected from a pool of individuals accompanying other patients visiting 2 local healthcare providers (health center no. 1 and no. 2, Havsa). The subjects were unrelated and had lived for 3 generations in the same geographical location. Informed consent was obtained for all study participants in accordance with institutional guidelines.

To assess the carrier rate of the mutations, we screened the subjects for the 4 FMF alleles M694V, V726A, M680I, and E148Q. Venous blood samples were collected from the subjects and preserved with ethylenediaminetetraacetic acid (EDTA). Genomic DNA was isolated from the blood samples using a Magna Pure LC High Pure PCR template preparation kit (Roche Diagnostics, Mannheim, Germany), and targeted PCR amplification of allelic regions, followed by restriction fragment length polymorphism (RFLP) analysis. The following specific primers were used: M694V: forward, 5′-AGAATGGCTACTGGGTGGTGAT-3′; reverse, 5′-AGAGAAAGAGCAGCTGGCGAATGTAT-3′. M680I: forward, 5′-GAAACAAGTGGGAGAGG-3′; reverse, 5′-CACCACCCAGTAGTAGCCATTCT-3′. V726A: forward, 5′-AGAATGGCTACTGGGTGGTG-3′; reverse, 5′-GCTGTGTTCTTCCCTCCATC-3′. E148Q: forward, 5′- AAACGGCACAGATGATTCCGCAG-3′; reverse, 5′-CCTTCTCTCTGCGTTTGCTCAGG -3′.

The restriction enzymes *Hinf*I, *Hph*I, *Alu*I, and *BstN*I were used for RFLP analysis of alleles M680I, M694V, V726A, and E148Q, respectively (Roche Diagnostics, Germany). All of these mutations were simultaneously tested with appropriate positive and negative controls. The positive results were repeated to ensure reproducibility. The PCR products and the digested fragments were electrophoresed on a 3% agarose gel and visualized by ethidium bromide staining.

### 2.1. Statistical analysis

The allele frequencies were compared to values cited in the literature for regions of Turkey with high FMF prevalence rates using the χ²- test.

## 3. Results

Table shows the distribution of MEFV variations among healthy populations from different regions of Turkey including Havsa [9-13]. We analyzed the allele frequencies and confidence intervals (CIs) for all the individuals.

**Table:** Frequencies of MEFV alleles among healthy populations in different regions of Turkey.

Region	n	M694V	E148Q	M680I	V726A
Havsa (current study)	263	8/526 (1.5%)	14/526 (2.6%)	3/526 (0.5%)	0/526
χ² test		6.21	9.78	9.85	0.00
p value		0.31	0.13	0.08	
Yigit9	100	8/200 (4%)	7/200 (3.5%)	5/200 (2.5%)	4/200 (2%)
Tunca10	49	3/98 (3%)	6/98 (6%)	0/98	2/98 (2%)
Cosan11	103	3/206 (1.5%)	7/206 (3.4%)	1/206 (0.5%)	1/206 (0.5%)
Yilmaz12	100	3/200 (1.5%)	12/200 (6%)	5/200 (2.5%)	2/200 (1%)
Imirzalioglu¹³	66	4/124 (3.2%)	1/130 (0.08%)	2/128 (1.5%)	0/132

The frequencies of the E148Q, M680I, M694V, and V726A mutations in the Havsa cohort were 2.6%, 0.5%, 1.5%, and 0.0%, respectively. All variations were heterozygous. One patient with FMF had been identified in a previous field study [8]; in the present study, this patient was found to be heterozygous for M694V. This patient was not included among the 263 statistically evaluated individuals.

## 4. Discussion

In this study, we investigated the frequencies of the 4 most common variants of the MEFV gene, i.e. M694V, V726A, M680I, and E148Q, observed in Turkish FMF patients among healthy subjects from the Havsa region. We previously reported that FMF prevalence in this part of the country is rather low compared to other regions (0.006%) (95% CI 0.005-0.007); the prevalence rates in other regions of Turkey are in the range of 0.027%-0.25% [8].

The primary criterion for the recruitment of the study cohort was to match the birthplace of the study individuals with that of the individuals reported in the previous study [8]. Our main aim was to investigate the mutation carrier rate in a population where FMF is less prevalent. We compared our frequency data to that reported in previous studies including all necessary data concerning the allelic and genotype frequencies of the 4 most common mutations in Turkish FMF patients.

A series of several interesting studies regarding the genetics of FMF in Turkey have been already published [6,7,12,14,15]. In two of the largest studies, namely by the Turkish FMF Study Group [6], and by Bilge et al. [7], the most common MEFV alleles were M694V (51.4% and 44.5% respectively) followed by M680I (14.4% and 12.3% respectively), V726A (8.6% and 9.2% respectively). Bilge et al. refer to E148Q as the fourth most common MEFV mutation (1%), while the Turkish FMF study group do not provide any data.

In the present study, our results suggest that among healthy individuals in Havsa, the difference between frequencies of the 4 mutational alleles is not significant (Table) when compared with the frequencies reported in studies conducted for the regions where FMF is more prevalent (χ²: 6.21, p: 0.28), [9-13]. In a study from İstanbul, which has the most heterogeneous population for MEFV mutations, a similar prevalence rate of M694V was observed in 103 healthy individuals (1.5% CI: 0.00-0.31 for İstanbul vs. 0.5-2.5 for Havsa) [11]. The M680I and E148Q allele frequencies were not significantly different from those found in the other studies (χ²: 9.85, p: 0.08 and χ²: 9.78, p: 0.13, respectively). The V726A mutation was not detected in the present study cohort, but this allele was not found in one of the other regions and statistical analysis could not be performed. Interestingly, in our previous study, which was conducted in the same region of Turkey, only 1 patient with FMF was identified, and he was heterozygous for the M694V allele [8].

Although the E148Q mutation is one of the most common mutations in patients with FMF, it occurs more frequently in the general population compared to FMF patients in the regions where the disease is prevalent. For example, one study indicated that the allele frequency was 3.5% among FMF patients vs. 6% in healthy Turkish subjects [12]. Consistent with our study results, Cosan et al. [11] reported that the E148Q mutation frequencies were higher than those of other mutations in healthy populations (2.6% [95% CI 1.6-3.9] and 3.4% [95% CI 0.9-5.9], respectively) (Table).

There are subtle and as yet, unexplained similarities in FMF allele frequencies observed in other countries. In Greece, frequencies of the common mutations mirror the prevalence rates observed in Eastern Mediterranean countries (M694V: 38.1%, M680I: 19.7%, V726A: 12.2%, and E148Q: 10.9%). However, the E148Q allele frequency among FMF patients in Greece is 10.9%, whereas it is 0% among the healthy population. Of note, the total MEFV mutation carrier rate among healthy individuals is relatively small (0.7%) in the healthy Greek population [16].

Some researchers think that FMF and Behçet’s disease (BD) have epidemiological similarities and have reported that some MEFV mutations are more frequent in BD patients compared to healthy controls [17,18]. As with FMF, we demonstrated that the prevalence of BD in Havsa was much lower (0.019%) compared to other regions of Turkey (2/10,000 in Havsa vs. 42/10,000, 11/10,000, and 37/10,000 in the prevalent regions) [8].

Despite the close association between HLA-B51 mutations and the pathogenesis of BD, the frequency of HLA-B51 in the healthy population in regions where BD is more prevalent is not significantly different from populations where BD is less prevalent [8]. The epidemiological similarities between these two diseases could be because of environmental or other genetic factors.

In this region, although there are similarities in the mutation frequencies, clinically diagnosed FMF patients are very rare. As previously reported, the pyrin gene penetrance displays different rates not only among ethnically different populations but also among regional groups of the same ethnicity [19]. More functional studies are required to determine the degree to which the aforementioned differences arise because of genetic or population-specific factors. One of the limitations of our study is the low statistical power for comparing the frequency of MEFV variants in healthy populations from different regions because of the low frequencies of some variants.

In conclusion, we think that the positivity of MEFV mutation tests have lower predictive value in a population with low prevalence. A correlation between clinical symptoms, MEFV mutations, and the geographical region of patients should be considered in the diagnosis of FMF patients.

## Ethics committee approval

Ethical approval was obtained from Marmara University Ethics Committee (B.30.2.MAR.0.01.02/AEK/108).
